# Supplementary material for: Analysis of PPARγ Signaling Activity in Psoriasis
Source: Int J Mol Sci. 2021 Aug 10;22(16):8603. doi: 10.3390/ijms22168603 (PMC8395241; doi:10.3390/ijms22168603)
Supplement: Supplementary file 1 [file ijms-22-08603-s001.zip › Supplemental materials_Analysis of PPARg signaling activity in psoriasis/Pathway models/Models images and html files/Interleukin-17 and interleukin-22 signaling in psoriasis/6307.html]

IKBKB


# Protein IKBKB

|  |  |
| --- | --- |
| URN | urn:agi-llid:3551 |
| Total Entities | 0 |
| Connectivity | 4287 |
| Name | IKBKB |
| Description | inhibitor of kappa light polypeptide gene enhancer in B-cells, kinase beta |
| Notes | The protein encoded by this gene phosphorylates the inhibitor in the inhibitor/NF-kappa-B complex, causing dissociation of the inhibitor and activation of NF-kappa-B. The encoded protein itself is found in a complex of proteins. Several transcript variants, some protein-coding and some not, have been found for this gene. [provided by RefSeq, Sep 2011] |
| Primary Cell Localization | Cytoplasm |
| Class | Protein kinase |
| ObjectType | Protein |

---

|  |  |
| --- | --- |
| Pathway | mTOR Signaling Hyperfunction |
|  | Toll-like Receptors Act through MYD88-TIRAP Signaling |
|  | Antiviral Signaling through Pattern Recognition Receptors |
|  | TLR4 Signaling in Leukocytes |
|  | Plasmin Effects in Inflammation |
|  | Toll-like Receptors in Sterile Inflammation |
|  | Prostaglandin E2 Receptor Signaling in Neurons |
|  | Macrophage M1 Lineage |
|  | AXL Receptor Inhibits Macrophages and Dendritic Cells Function |
|  | SCF/FBXO7 Complex |
|  | Myocardial Remodeling |
|  | Myocardial Infarction |
|  | MAVS in Antiviral Innate Immune Response in Myocarditis |
|  | Toll-like Receptors (TLR) in Antiviral Innate Immune Response |
|  | Apoptotic Keratinocytes Clearance Recession in Systemic Lupus Erythematosus |
|  | Dendritic Cell Activation in Systemic Lupus Erythematosis |
|  | B-Cell Activation in Systemic Lupus Erythematosus |
|  | Humoral Immunity in Vitiligo |
|  | Plasmacytoid Dendritic Cell in Diabetes Mellitus Type 1 |
|  | Toll-like Receptors in beta-Cell |
|  | Conventional Dendritic Cell Role in Diabetes Mellitus Type 1 |
|  | Proteins Involved in Diabetes Mellitus Type 2 |
|  | AGE Related Insulin Resistance in Skeletal Muscles |
|  | Proteins Involved in Insulin Resistance |
|  | Insulin Resistance in Myocytes Induced by Obesity |
|  | Adipose Tissue Inflammation Provokes Insulin Resistance |
|  | Proteins Involved in non-Alcoholic Fatty Liver Disease |
|  | beta-Cell Destruction through Cytokines in Diabetes Mellitus |
|  | SOCS3 and PTPN1 in Hypothalamic Neuron Insensitivity to Insulin and Leptin |
|  | Enterocyte Mediated Mucosal Damage in Celiac Disease |
|  | Celiac Disease |
|  | OipA Signaling in Helicobacter Pylori Infection |
|  | Proteins Involved in Helicobacter Infections |
|  | CagA Phosphorylation Independent Signaling |
|  | Dendritic Cell Function in Ulcerative Colitis |
|  | Nicotine anti-Inflammatory Effect in Ulcerative Colitis |
|  | Splenic Marginal Zone Lymphoma |
|  | TSLP Signaling in Bronchial Epithelial Cell in Asthma |
|  | Club-Cell Role in Asthma |
|  | Antigen-Presenting Cell Role in Asthma |
|  | Osteoclast Activation in Gout |
|  | Monocytes Function in Gout |
|  | Dendritic Cell Dysfunction in Psoriatic Arthritis |
|  | Osteoclast Activation in Psoriatic Arthritis |
|  | Osteoclast Activation in Rheumatoid Arthritis |
|  | TLR2 Induced Synovial Fibroblast Activation in Rheumatoid Arthritis |
|  | B-Cells Function in Systemic Scleroderma |
|  | Toll-like Receptors (TLR) in Periodontitis |
|  | Diabetes Induced Periodontitis |
|  | IL1 Receptor Antagonist (IL1RN) Deficiency (DIRA) |
|  | PAPA Syndrome |
|  | Mevalonate Kinase Deficiency |
|  | Blau Syndrome |
|  | Cryopyrin (NLPR3) Associated Periodic Syndromes |
|  | Familial Cold Autoinflammatory Syndrome 2 |
|  | Familial Mediterranean Fever |
|  | Majeed Syndrome |
|  | NF-kB Signaling Activation in Huntington Disease |
|  | Huntingtin Mutant Protein Toxicity Effects |
|  | Gluten Impact on Neuronal System (Hypothesis) |
|  | Osteoclasts Function in Osteopetrosis |
|  | Osteoclast Activation in Postmenopause |
|  | HMGB1 and IL1B in Neuroinflammation |
|  | Crystallin alpha B (CRYAB) Suppresses Multiple Sclerosis |
|  | SPP1 Induced Survival of Myelin Specific T-Cells in Multiple Sclerosis |
|  | HPV E6 and E7 Induced Disruption of TNFR/NF-kB Signaling in Keratinocytes |
|  | OPTN Effects on NF-kB Signaling in Glaucoma |
|  | Immune System Role in Otitis Media |
|  | Plasmin Role in Glomerulonephritis |
|  | Endothelial Cell Dysfunction in Glomerulonephritis |
|  | Toll-like Receptors in Pyelonephritis |
|  | Endothelial Cell Dysfunction in Pyelonephritis |
|  | Genes with Mutations in Cancer-Associated Sustaining of Proliferative Signaling |
|  | NFKB Canonical Signaling Activation in Cancer |
|  | STAT3 and NFkB Activate Inflammation-Induced Tumorigenesis |
|  | IL23A and IL17A Provoke Cancer-Associated Inflammation |
|  | NFKB Signaling Activation by Blocking of Tumor Suppressors |
|  | Apoptosis Block by Death Receptor Signaling in Cancer |
|  | Apoptosis Evasion in Cancer: Overview |
|  | Telomeric Proteins/NFKB Positive Feedback Interplay in Cancer |
|  | Oxidized LDL/OLR1 Activates Cell Proliferation in Cancer |
|  | ANGPT/TEK Maintains Endothelial Cell Quiescence in Cancer |
|  | Treg Cells Promote Immunosuppression in Cancer Immune Escape |
|  | IDO1 in Cancer Immune Escape |
|  | Effector T-cell Inactivation in Cancer Immune Escape |
|  | IFNLR1-> STAT/NFKB Signaling |
|  | NF-kB Canonical Signaling |
|  | T-Cell Receptor -> NF-kB Signaling |
|  | pathway 4 |
|  | sars-cov binding |
|  | dsRNA induces innate immunity and INFs in alveolar cells |
|  | Dendritic Cell Activation in COVID19 |
|  | 1\_Inflammation in keratinocytes in the pathology of acne |
|  | 2\_Interleukin-17 and interleukin-22 signaling in psoriasis |
|  | 1\_The onset of atopic dermatitis |
|  | 1\_TNF and IL1B provoke ECM degradation in osteoarthritis |
|  | 1\_Osteoclast-mediated bone resorption in postmenopausal women |
|  | 1\_Osteoclast functional impairment in osteopetrosis |
|  | 2\_Chondrocyte death in osteoarthritis |
|  | 1\_The role of Toll-like receptors in pyelonephritis |
|  | 2\_The role of endothelial cells in pyelonephritis |
|  | 1\_Insufficient activation of immune response in the middle ear epithelium cells in otitis media |
|  | 2\_Pathogens stimulate mucins expression in the middle ear |
|  | 2\_Oxidized LDL causes endothelial dysfunction in atherosclerosis |
|  | 1\_Cigarette smoke causes dysfunction and production of inflammatory mediators in alveolar epithelial cells |
|  | 3\_Role of alveolar macrophages in pulmonary tissue destruction in COPD |
|  | 1\_Th2 cell response in asthma |
|  | 3\_2\_Regulation of airway surface liquid (ASL) thickness |
|  | 3\_1\_Mucus accumulation in asthma |
|  | 2\_2\_Eosinophilia and neutrophilia in asthma: eosinophil apoptosis |
|  | 4\_Mucin hyperproduction in goblet and mucous cells in COPD |
|  | 2\_Paneth cell dysfunction in Crohn's disease |
|  | 2\_Intestine epithelial cells drive immunological responses in ulcerative colitis |
|  | 1\_Intestine epithelial cell dysfunction in ulcerative colitis |
|  | 2\_Lipotoxicity in NAFLD |
|  | 1\_CD4+ T cell and epithelial cell response in celiac disease |
|  | 6\_p12I Viral protein promotes T-cell activation in HTLV-1 infection |
|  | 5\_Human papilloma virus E6 and E7 proteins promote epithelial cell proliferation |
|  | 2\_HIV - induced depletion of CD4+ T-cells |
|  | 4\_2\_Insulin resistance: inflammation-related insulin resistance |
|  | 4\_3\_Insulin resistance: FFA-related insulin resistance |
|  | 1\_CFTR expression and degradation in epithelial cells in cystic fibrosis (class I-II mutations) |
|  | 1\_Antigen-presenting cells promote islet dysfunction and immune system activation in T1D |
|  | 2\_Neuron and Schwann cell death |
|  | Figure 4\_Diabetes mellitus Type 1: conventional dendritic cell |
|  | Interleukin-17 and interleukin-22 signaling in psoriasis |

---

|  |  |
| --- | --- |
| Group | Genes with Mutations Associated with Ageing (Mammalian Animal Model) |
|  | Genes with Mutations Associated with Splenic Marginal Zone Lymphoma |
|  | binding partnetrs for SARSs |

---

|  |  |
| --- | --- |
| MedScan ID | 3551 |

---

|  |  |
| --- | --- |
| LocusLink ID | 3551 |
|  | 16150 |
|  | 84351 |

---

|  |  |
| --- | --- |
| Alias | IKK2 |
|  | Nuclear factor NF-kappa-B inhibitor kinase beta |
|  | inhibitor of kappa light polypeptide gene enhancer in B-cells kinase beta |
|  | NFKBIKB |
|  | FLJ33771 |
|  | AI132552 |
|  | I-kappa-B kinase II |
|  | IkB kinase II |
|  | inhibitor of kappa light polypeptide gene enhancer in B-cells, k |
|  | inhibitor of kappaB kinase beta |
|  | IMD15B |
|  | IkB kinase beta |
|  | NFKBIKBs |
|  | Inhibitor of nuclear factor kappa B kinase beta subunit |
|  | IKKB |
|  | FLJ40509 |
|  | nuclear factor kappa B inhibitor kinase beta |
|  | IKK-B |
|  | inhibitor of nuclear factor kappa-B kinase subunit beta |
|  | NF kappa B inhibitor kinase beta |
|  | IMD15A |
|  | IKBKB |
|  | IKKbeta |
|  | FLJ38368 |
|  | OTTMUSP00000022456 |
|  | FLJ36218 |
|  | IKK[b] |
|  | inhibitor of kappa light polypeptide gene enhancer in B-cells, kinase |
|  | MGC131801 |
|  | I kappaB kinase beta |
|  | IkB kinase 2 |
|  | I-kappa-B kinase 2 |
|  | IMD15 |
|  | IKK-beta |
|  | I-kappa-B-kinase beta |
|  | inhibitor of kappa light polypeptide gene enhancer in B-cells, kinase beta |
|  | IKK-2 |
|  | AIM-1 |

---

|  |  |
| --- | --- |
| EC Number | 2.7.11.10 |

---

|  |  |
| --- | --- |
| GO ID | 0005524 |
|  | 0008384 |
|  | 0046982 |
|  | 0042803 |
|  | 0004672 |
|  | 0019901 |
|  | 0004674 |
|  | 0097110 |
|  | 0038095 |
|  | 0007249 |
|  | 0007252 |
|  | 0002756 |
|  | 0050852 |
|  | 0035666 |
|  | 0002479 |
|  | 0071356 |
|  | 0030866 |
|  | 0006954 |
|  | 0045087 |
|  | 0070498 |
|  | 0043066 |
|  | 1903347 |
|  | 0035509 |
|  | 0018105 |
|  | 0043123 |
|  | 0051092 |
|  | 0045944 |
|  | 0045893 |
|  | 0072659 |
|  | 0006468 |
|  | 1903140 |
|  | 0042325 |
|  | 0010803 |
|  | 0009615 |
|  | 0002223 |
|  | 0051403 |
|  | 0033209 |
|  | 0016032 |
|  | 0035631 |
|  | 0008385 |
|  | 0005737 |
|  | 0009898 |
|  | 0005829 |
|  | 0045121 |
|  | 0005634 |
|  | 0016301 |
|  | 0000166 |
|  | 0019903 |
|  | 0016740 |
|  | 0001782 |
|  | 0031175 |
|  | 0016310 |
|  | 2001259 |
|  | 0008284 |
|  | 1901216 |
|  | 0010976 |
|  | 0010765 |
|  | 0061847 |
|  | 0043231 |
|  | 0016020 |
|  | 0042493 |
|  | 0070542 |
|  | 0032496 |
|  | 0009636 |
|  | 0003009 |
|  | 0034146 |
|  | 0006916 |
|  | 0035872 |
|  | 0034142 |
|  | 0032481 |
|  | 0002755 |
|  | 0023014 |
|  | 0004702 |
|  | 0005515 |
|  | 0034134 |
|  | 0034138 |
|  | 0042501 |
|  | 0008063 |
|  | 0034130 |
|  | 0048011 |
|  | 0034166 |
|  | 0070423 |
|  | 0038123 |
|  | 0016772 |
|  | 0038124 |
|  | 0008152 |
|  | 0034162 |
|  | 0043122 |
|  | 0090002 |
|  | 0009653 |
|  | 0002224 |
|  | 0016563 |
|  | 0005792 |

---

|  |  |
| --- | --- |
| KEGG ID | hsa:3551 |
|  | mmu:16150 |
|  | rno:84351 |

---

|  |  |
| --- | --- |
| Organism | Homo sapiens {Organism urn:agi-taxid:9606} |
|  | Mus musculus {Organism urn:agi-taxid:10090} |
|  | Rattus norvegicus {Organism urn:agi-taxid:10116} |
|  | Homo sapiens |
|  | Mus musculus |
|  | Rattus norvegicus |

---

|  |  |
| --- | --- |
| Mouse chromosome position | 8 |

---

|  |  |
| --- | --- |
| OMIM ID | 603258 |
|  | 615592 |
|  | 618204 |

---

|  |  |
| --- | --- |
| Rat chromosome position | 16q12.5 |

---

|  |  |
| --- | --- |
| Hugo ID | 5960 |
|  | HGNC:5960 |

---

|  |  |
| --- | --- |
| Human chromosome position | 8p11.21 |
|  | 8p11.2 |

---

|  |  |
| --- | --- |
| Swiss-Prot Accession | O14920 |
|  | O14920.1 |
|  | Q5D0E0 |
|  | A0A0R4J0T4 |
|  | O88351 |
|  | O88351.1 |
|  | G3V8H5 |
|  | Q9QY78 |
|  | Q9QY78.1 |
|  | B4DZ30 |
|  | B4E0U4 |
|  | O75327 |
|  | Q9R1J6 |
|  | B4DP95 |
|  | Q32ND9 |
|  | Q59GL9 |
|  | Q05DR8 |
|  | Q3U141 |
|  | Q3USX8 |
|  | Q3V454 |
|  | Q8BQA9 |
|  | E9PH46 |

---

|  |  |
| --- | --- |
| GenBank ID | NC\_000008 |
|  | XR\_949402 |
|  | NR\_033818 |
|  | NR\_033819 |
|  | NR\_040009 |
|  | XR\_001745530 |
|  | XM\_005273491 |
|  | XP\_005273548 |
|  | XM\_005273496 |
|  | XP\_005273553 |
|  | XM\_005273493 |
|  | XP\_005273550 |
|  | XM\_005273498 |
|  | XP\_005273555 |
|  | NM\_001190720 |
|  | NP\_001177649 |
|  | NM\_001556 |
|  | NP\_001547 |
|  | NM\_001242778 |
|  | NP\_001229707 |
|  | XM\_011544518 |
|  | XP\_011542820 |
|  | XM\_005273492 |
|  | XP\_005273549 |
|  | XM\_005273490 |
|  | XP\_005273547 |
|  | XM\_005273494 |
|  | XP\_005273551 |
|  | XM\_017013396 |
|  | XP\_016868885 |
|  | XM\_011544521 |
|  | XP\_011542823 |
|  | XM\_011544522 |
|  | XP\_011542824 |
|  | XM\_005273495 |
|  | XP\_005273552 |
|  | XM\_011544517 |
|  | XP\_011542819 |
|  | XM\_011544520 |
|  | XP\_011542822 |
|  | XM\_011544519 |
|  | XP\_011542821 |
|  | NG\_041793 |
|  | AC083973 |
|  | AY663108 |
|  | AAT65965 |
|  | CH471080 |
|  | EAW63222 |
|  | EAW63223 |
|  | EAW63224 |
|  | EAW63225 |
|  | EAW63226 |
|  | KF458748 |
|  | KF495673 |
|  | AB209090 |
|  | BAD92327 |
|  | AF029684 |
|  | AAC51860 |
|  | AF031416 |
|  | AAC64675 |
|  | AF080158 |
|  | AAD08997 |
|  | AI379342 |
|  | AK091090 |
|  | AK091297 |
|  | BAG52329 |
|  | AK093537 |
|  | AK095687 |
|  | AK097828 |
|  | AK290952 |
|  | BAF83641 |
|  | AK298241 |
|  | BAG60507 |
|  | AK302723 |
|  | BAG63942 |
|  | AK303294 |
|  | BAG64369 |
|  | AK303528 |
|  | BAG64556 |
|  | AK316418 |
|  | BAH14789 |
|  | AL708460 |
|  | BC006231 |
|  | AAH06231 |
|  | BC090865 |
|  | BC108694 |
|  | AAI08695 |
|  | BC127663 |
|  | AAI27664 |
|  | BC141978 |
|  | AAI41979 |
|  | BC144080 |
|  | BX648165 |
|  | DA043516 |
|  | DA105584 |
|  | DA159382 |
|  | DA709000 |
|  | O14920 |
|  | NC\_000074 |
|  | NM\_001159774 |
|  | NP\_001153246 |
|  | NM\_010546 |
|  | NP\_034676 |
|  | XM\_006509019 |
|  | XP\_006509082 |
|  | XM\_006509020 |
|  | XP\_006509083 |
|  | XM\_006509021 |
|  | XP\_006509084 |
|  | XM\_006509022 |
|  | XP\_006509085 |
|  | XM\_006509023 |
|  | XP\_006509086 |
|  | AC121835 |
|  | CH466580 |
|  | EDL32880 |
|  | EDL32881 |
|  | AA203913 |
|  | AF026524 |
|  | AAC23557 |
|  | AF088910 |
|  | AAD52095 |
|  | AK003927 |
|  | AK011359 |
|  | BAE43224 |
|  | AK051128 |
|  | BAC34533 |
|  | AK053346 |
|  | AK086442 |
|  | AK139972 |
|  | BAE24202 |
|  | AK144048 |
|  | AK144603 |
|  | BAE25962 |
|  | AK150884 |
|  | BAE29931 |
|  | AK151888 |
|  | AK151955 |
|  | BAE30827 |
|  | AK153151 |
|  | BAE31761 |
|  | AK155587 |
|  | AK156294 |
|  | BAE33659 |
|  | AK172275 |
|  | BAE42920 |
|  | AK177348 |
|  | AK182153 |
|  | AK186184 |
|  | AK194273 |
|  | AK201390 |
|  | AK219506 |
|  | BC004772 |
|  | AAH04772 |
|  | BC019210 |
|  | BC020334 |
|  | BC037723 |
|  | AAH37723 |
|  | BY015258 |
|  | O88351 |
|  | NC\_005115 |
|  | NM\_053355 |
|  | NP\_445807 |
|  | AC\_000084 |
|  | AABR07026361 |
|  | AAHX01088838 |
|  | AAHX01088839 |
|  | AAHX01088840 |
|  | CH473970 |
|  | EDM09011 |
|  | EDM09012 |
|  | AF115282 |
|  | AAF21978 |
|  | CB810812 |
|  | CO383061 |
|  | Q9QY78 |
|  | XM\_005273500 |
|  | XR\_870200 |
|  | AABR06089167 |
|  | ABBA01021152 |
|  | ABBA01021153 |
|  | AMYH02017890 |
|  | AC\_000030 |
|  | AC\_000140 |
|  | AAHY01068478 |
|  | AAHY01068479 |
|  | XP\_005273556 |
|  | XP\_005273557 |
|  | XP\_005273554 |
|  | NC\_018919 |
|  | XM\_005273497 |
|  | XM\_005273499 |
|  | NR\_033820 |
|  | NM\_001190722 |
|  | NP\_001177651 |
|  | NM\_001190721 |
|  | NP\_001177650 |
|  | NT\_167187 |
|  | NW\_001839130 |
|  | NW\_923907 |
|  | AC\_000051 |
|  | CR607076 |
|  | Q32ND9 |
|  | Q59GL9 |
|  | NT\_039457 |
|  | NW\_001030892 |
|  | Q05DR8 |
|  | Q3U141 |
|  | Q3USX8 |
|  | Q3V454 |
|  | Q5D0E0 |
|  | Q8BQA9 |
|  | NW\_047475 |
|  | NW\_001084719 |

---

|  |  |
| --- | --- |
| Swiss-Prot ID | IKKB\_HUMAN |
|  | IKKB\_MOUSE |
|  | IKKB\_RAT |
|  | Q32ND9\_HUMAN |
|  | B4DP95\_HUMAN |
|  | B4DZ30\_HUMAN |
|  | E9PH46\_HUMAN |
|  | Q5D0E0\_MOUSE |

---

|  |  |
| --- | --- |
| Cell Localization | Cytoplasm |
|  | Nucleus |
|  | Membrane raft |

---

|  |  |
| --- | --- |
| Ensembl ID | ENSG00000104365 |
|  | ENSP00000404920.3 |
|  | ENST00000416505.6 |
|  | ENSP00000430684.1 |
|  | ENST00000520810.6 |
|  | ENST00000342222.6 |
|  | ENST00000629753.1 |
|  | ENSP00000428186.1 |
|  | ENST00000521661.5 |
|  | ENSMUSG00000031537 |
|  | ENSMUSP00000033939.6 |
|  | ENSMUST00000033939.12 |
|  | ENSMUSP00000064235.3 |
|  | ENSMUST00000063401.9 |
|  | ENSMUSP00000138378.1 |
|  | ENSMUST00000135326.7 |
|  | ENSRNOG00000019073 |
|  | ENSRNOP00000025851.2 |
|  | ENSRNOT00000025851.4 |
|  | ENST00000629753 |
|  | ENSMUST00000125314 |
|  | ENST00000416505 |
|  | ENSP00000430868 |
|  | ENST00000521661 |
|  | ENST00000520810 |
|  | ENSP00000404920.2 |
|  | ENSP00000430868.1 |
|  | ENST00000520835 |
|  | ENST00000342222 |
|  | ENSMUSP00000138378 |
|  | ENST00000416505.5 |
|  | ENST00000520810.5 |
|  | ENSP00000430684 |
|  | ENST00000520835.5 |
|  | ENSP00000404920 |
|  | ENSRNOP00000025851 |
|  | ENSMUST00000135326 |
|  | ENSRNOT00000025851 |
|  | ENSP00000428186 |
|  | ENSMUST00000033939 |
|  | ENSMUST00000063401 |
|  | ENSMUSP00000033939 |
|  | ENSMUSP00000064235 |

---

|  |  |
| --- | --- |
| MGI ID | MGI:1338071 |
|  | 1338071 |

---

|  |  |
| --- | --- |
| RGD ID | 621375 |

---

|  |  |
| --- | --- |
| Unigene ID | Rn.19222 |
|  | Mm.277886 |
|  | Hs.597664 |

---

|  |  |
| --- | --- |
| Homologene ID | 7782 |

---

|  |  |
| --- | --- |
| Shape | Sickle-vertex |

---

|  |  |
| --- | --- |
| IPI ID | IPI00213608 |
|  | IPI00024709 |
|  | IPI00798101 |
|  | IPI00982033 |
|  | IPI00130038 |
|  | IPI01010552 |
|  | IPI00656051 |
|  | IPI00981929 |
|  | IPI00556772 |
|  | IPI00556516 |
|  | IPI00796308 |
|  | IPI00789633 |
|  | IPI00654351 |
|  | IPI00653754 |
|  | IPI00458320 |
|  | IPI00830374 |
|  | IPI00831429 |
|  | IPI00782738 |

---
